# Doxycycline for Malaria Chemoprophylaxis and Treatment: Report from the CDC Expert Meeting on Malaria Chemoprophylaxis

**DOI:** 10.4269/ajtmh.2011.10-0285

**Published:** 2011-04-05

**Authors:** Kathrine R. Tan, Alan J. Magill, Monica E. Parise, Paul M. Arguin

**Affiliations:** Division of Parasitic Diseases and Malaria, Center for Global Health, Centers for Disease Control and Prevention, Atlanta, Georgia; Walter Reed Army Institute for Research, Silver Spring, Maryland

## Abstract

Doxycycline, a synthetically derived tetracycline, is a partially efficacious causal prophylactic (liver stage of *Plasmodium*) drug and a slow acting blood schizontocidal agent highly effective for the prevention of malaria. When used in conjunction with a fast acting schizontocidal agent, it is also highly effective for malaria treatment. Doxycycline is especially useful as a prophylaxis in areas with chloroquine and multidrug-resistant *Plasmodium falciparum* malaria. Although not recommended for pregnant women and children < 8 years of age, severe adverse events are rarely reported for doxycycline. This report examines the evidence behind current recommendations for the use of doxycycline for malaria and summarizes the available literature on its safety and tolerability.

## Summary

### Indications.

The only U.S. Food and Drug Administration (FDA)-approved indication for the use of doxycycline for malaria is for prophylaxis of *Plasmodium falciparum* in short-term travelers (< 4 months) to areas with chloroquine and/or pyrimethamine-sulfadoxine-resistant strains. Although not FDA approved, doxycycline is recommended for prophylaxis of other species of malaria^1^ and may be used long term. When used in conjunction with other medications, doxycycline can be used for the treatment of malaria in non-pregnant adults and children ≥ 8 years of age: with quinine sulfate for uncomplicated, chloroquine-resistant *P. falciparum*; with both quinine sulfate and primaquine for uncomplicated chloroquine-resistant *Plasmodium vivax*; and with parenteral quinidine for severe malaria.^2^

### Dosing.

Available in multiple dosage forms and strengths, doxycycline tablets or capsules of 100 mg are most often used for malaria prophylaxis or treatment. Tablets or capsules should be swallowed with an adequate amount of fluid and should be taken with food. Intravenous (IV) formulation is available for patients unable to take oral medications for treatment. For IV, avoid rapid administration.

#### Adults.

Prophylaxis: 100 mg base once daily starting 1–2 days before travel, then daily during travel, and daily for 4 weeks after leaving the malarious area.

Treatment: 100 mg twice a day for 7 days; must be used in conjunction with a fast acting schizontocide. Primaquine is also required if treating *P. vivax* or *Plasmodium ovale*.

#### Children.

For children ≥ 8 years of age.

Prophylaxis: 2.2 mg/kg (up to adult dose of 100 mg) daily starting 1–2 days before travel, daily during travel, and daily for 4 weeks after leaving the malarious area.

Treatment: 2.2 mg/kg (up to adult dose of 100 mg) twice a day for 7 days; must be used in conjunction with a fast acting schizontocide. Primaquine is also required if treating *P. vivax* or *P. ovale*.

### Efficacy.

Prophylaxis: Protective efficacy of doxycycline has been shown in three randomized placebo-controlled trials to be between 92% and 96% for *P. falciparum* and 98% for primary *P. vivax* infection.

Treatment: When used in combination with a fast acting schizontocide, treatment efficacy of doxycycline has been shown to be 96–100% in three open-label trials.

### Pharmacokinetics.

Limited data suggest that milk and other dairy products may limit the bioavailability of doxycycline. It is recommended to separate doxycycline and ingestion of dairy products by 2–3 hours.

### Adverse drug reactions.

Most common mild/moderate adverse drug reactions (ADRs): nausea, vomiting, abdominal pain, photosensitivity, and vaginitis. Women prone to candidal vaginitis should consider carrying a self-treatment course of an anti-fungal. Severe ADRs are uncommon and include esophagitis and esophageal ulcerations. Doxycycline monohydrate has been observed to have less gastrointestinal side effects than doxycycline hyclate.

### Contraindications.

Doxycycline is contraindicated in those who have known hypersensitivity to tetracyclines.

### Drug interactions.

Decreased absorption of doxycycline can occur if taken concurrently with medications with divalent or trivalent cations such as antacids, laxatives, and oral iron preparations. Although the current FDA label states “concurrent use of tetracycline may render oral contraceptives less effective,” recent studies have failed to show a significant association between oral contraceptive failure and tetracyclines. Tetracyclines may potentiate the effect of oral anticoagulants, therefore patients on oral anticoagulants should have prothrombin times monitored closely and dose adjusted as needed. Concurrent use of tetracyclines and methoxyflurane anesthesia is not recommended. Barbiturates, carbamazepine, and phenytoin decrease the half-life of doxycycline.

### Warnings.

Pregnancy category D: Do not use in pregnancy except for the treatment of life-threatening multidrug-resistant *P. falciparum* when no other treatment options are available. Not recommended for children < 8 years of age.

### Use during breastfeeding.

Excreted in low concentrations in breast milk; noted to be compatible with breastfeeding by the American Academy of Pediatrics.

## Introduction

Doxycycline is a broad-spectrum bacteriostatic agent synthetically derived from naturally occurring tetracyclines produced by *Streptomyces* sp. bacteria.^3^ Doxycycline was invented and clinically developed in the early 1960s by Pfizer Inc., New York, NY, and marketed under the brand name Vibramycin. Vibramycin received FDA approval in 1967, becoming Pfizer's first once-a-day broad-spectrum antibiotic. The additional indication for malaria prophylaxis was obtained in 1994 with a supplemental New Drug Application. Tetracyclines were first reported efficacious against drug-resistant malaria in the 1970s.^4^,^5^ Early studies examined the effectiveness of tetracycline, minocycline, and doxycycline as antimalarials.^4^–^8^ The evidence for minocycline beyond these early studies is limited, and the four times per day dosing of tetracycline may make adherence difficult. Of the tetracyclines, doxycycline has the most evidence for its effectiveness as an antimalarial.

## Recommended Uses and Dosing of Doxycycline

Doxycycline can be used by travelers to all malaria-endemic areas for malaria prophylaxis. When used in conjunction with other medications, doxycycline can also be used to treat malaria.

### Prophylaxis.

Doxycycline can be used for the prevention of malaria in travelers to malaria-endemic areas and is a good option for areas with chloroquine or multidrug-resistant *P. falciparum*. For prophylaxis, doxycycline is taken once daily beginning 1–2 days before travel, while in malarious areas, and for 4 weeks after leaving. Daily dosage for children ≥ 8 years of age is 2.2 mg/kg (not to exceed the adult dose of 100 mg) and for adults 100 mg.

### Treatment.

Doxycycline should always be used in conjunction with another drug for treatment of malaria. Quinine with doxycycline can be used for uncomplicated malaria caused by chloroquine-resistant *P. falciparum* or *P. vivax*. When used for severe malaria doxycycline should be used with intravenous quinidine. The treatment dose of doxcycyline in adults is 100 mg twice a day and for children 2.2 mg/kg twice a day up to a maximum of 100 mg twice daily. For those unable to tolerate oral medications, doxycycline is available in a parenteral form and can be made in concentrations between 0.1 and 10 mg/mL. If pain occurs while receiving doxycycline intravenously, it is recommended to use more dilute concentrations (no lower than 0.1 mg/mL) infused at rates no higher than 1 mg/min^9^; switch to an oral formulation once the patient is able to tolerate medications by mouth. If treating *P. vivax* or *P. ovale*, in addition to the aforementioned drugs, primaquine must also be given to prevent relapses (see Pharmacodynamics section).

## Efficacy and Effectiveness

The emergence of chloroquine-resistant *P. falciparum* in the 1960s spurred efforts to examine alternative medications such as tetracyclines with antimalarial activity. Early studies examined the treatment and prophylactic efficacy of tetracyclines such as doxycycline, tetracycline, and minocycline against chloroquine-resistant *P. falciparum*. Since these earlier studies, the preponderance of literature on efficacy of tetracyclines as antimalarials has focused on doxycycline.

Overall, studies have showed that doxycycline is a slow-acting blood schizontocidal agent, had partial causal effects and no sporontocidal or gametocytocidal effects, and is effective in the treatment and prevention of malaria (prophylaxis Table 1).^10^–^18^ Doxycycline 100 mg daily has been shown to be highly effective as a blood schizonticidal agent, meaning that it kills the asexual, erythrocytic stages of the malaria parasite.^10^,^12^–^21^ Three randomized, double-blinded, placebo-controlled studies showed that in semi-immune patients, 100 mg of doxycycline daily had a protective efficacy of 84% (95% confidence interval [CI] = 79.9–97.5%), 92.6% (95% CI = 79.9–97.5%), and 96% (95% CI = 85.4–99.6%) for *P. falciparum* infections.^10^,^11^,^18^ One of these studies found that doxycycline had a 98% (95% CI = 88.0–99.9%) protective efficacy against primary *P. vivax* infection,^18^ but did not examine protective efficacy against relapses. Another randomized, double-blinded, placebo-controlled study showed a 99% (95% CI = 94–100%) protective efficacy of doxycycline 100 mg daily for *P. falciparum* in 67 non-immune Indonesian soldiers.^13^ However, in two of these studies, participants were not followed up after leaving the malaria-endemic areas, and it was unclear if prophylactic medications were continued after leaving. One open-label trial followed non-immune patients for 3 months after returning from a malaria-endemic country. Patients had received 100 mg of daily doxycycline while abroad. Of these 171 patients, only 5 (0.6%) developed *P. falciparum* malaria during the follow-up period. Of these 5, one developed malaria while on prophylaxis and 4 developed symptoms after discontinuation of prophylaxis during the 3 months of observation upon returning to a malaria-free country.^12^

Other studies have also shown doxycycline to have inadequate causal activity, or activity against the liver stage of the malaria parasite, which has implications for doxycycline's post-exposure course.^5^,^22^–^24^ One randomized, double-blinded, placebo-controlled trial examined the efficacy of doxycycline prophylaxis for *P. falciparum* malaria when taken by individuals exposed to mosquitoes infected with *P. falciparum*. Doxycycline was taken daily 3 days before exposure and for 6 days post exposure. Four of 12 persons taking that preventive regimen developed falciparum malaria 15–24 days after completing medications; the protective efficacy was 67% (95% CI = 35–90%).^23^ Another study found that of 60 soldiers who received 100 mg of doxycycline a day before deployment, 6 weeks during their deployment, and for 3 days after their return, none had falciparum malaria, whereas two developed vivax malaria.^16^ These studies suggest that doxycycline has some tissue schizontocidal activity for *P. falciparum*, but is insufficient for causal prophylaxis. Doxycycline would therefore have to be continued for a sufficient amount of time post exposure for successful suppression of parasites that emerge from the liver. Studies have observed that^5^,^22^–^24^ malaria developed within 3–4 weeks post exposure. It is therefore recommended that doxycycline prophylaxis be continued for 4 weeks post exposure.

Doxycycline's limited activity in the liver stages of *Plasmodium* also means that it does not kill *P. vivax* hypnozoites. Although very effective for preventing primary *P. vivax* infections, doxycycline is not effective at preventing relapses.

The dose of doxycycline required to achieve successful malaria prophylaxis was also examined. The efficacy of half-dose doxycycline (50 mg/day, *N* = 67) was compared with full-dose doxycycline (100 mg/day, *N* = 77). One open-label, randomized, controlled trial found that full-dose doxycycline was significantly more effective in prevention of *P. vivax,* however the study was not powered to detect a difference between the two doses of doxycycline in preventing *P. falciparum* infections.^14^ Another study examined the effectiveness of full-dose and half-dose doxycycline under operational conditions; adherence to medications was not enforced. Although adherence to full versus half-dose doxycycline was not examined, the study found that on average 73% of the doxycycline regimen was taken and that half-dose (33% infected) was significantly less effective than full-dose doxycycline (19% infected) at preventing both *P. falciparum* and primary *P. vivax* infection.^17^

### Treatment (Table 2)^5^,^26^–^29^.

Doxycycline has been shown to be a slower acting blood schizonticide during the course of an acute *Plasmodium* infection relative to other antimalarials.^30^ However, doxycycline achieves good treatment efficacy when given with rapidly acting shizontocidal drugs such as quinine or quinidine. Studies have reported parasite clearance times (*P. falciparum*) between 3.8 and 6.0 days and fever clearance times between 3.6 and 4.0 days in patients treated with only tetracycline or doxycycline.^4^,^7^,^25^,^26^ These clearance times are longer than those observed in patients given quinine alone with parasite and fever clearance times of 2.6 days and 1.9–2.6 days, respectively.^31^,^32^ In terms of treatment efficacy, in patients given doxycycline or tetracycline alone for *P. falciparum*, cure rates between 75% and 100% have been reported.^5^,^7^ Therefore, the slowness with which tetracyclines act make them of limited usefulness as a monotherapy in *P. falciparum* infections.^5^,^7^ The usefulness of tetracycline when given with a faster acting schizontocidal drug, quinine, was studied when decreased effectiveness of quinine was observed in Southeast Asia.^7^,^32^,^33^ Two open-label trials, one non-randomized and the other randomized, gave subjects quinine (640 mg three times daily for 3 days, and 600 mg four times daily for 7 days, respectively) and tetracycline (250 mg four times daily for 10 days) and found treatment efficacies between 96% and 98% with average parasite clearance times between 3 and 3.6 days and a fever clearance time of 2.6 days.^7^,^34^ Furthermore, one study observed that tetracycline contributed to the maintenance of plasma quinine levels above the minimum inhibitory concentration during the treatment period.^35^ It was then extrapolated from these studies that quinine plus doxycycline would also be an effective malaria treatment regimen. More recently, an open-label trial showed 100% cure of all subjects given quinine (10 mg/kg three times a day for 3 to 7 days) and doxycycline (100 mg twice a day for 7 days) for treatment of uncomplicated *P. falciparum* malaria.^29^ Therefore, doxycycline should be given with a rapidly acting schizontocide for timely and effective malaria treatment.

## Pharmacokinetics and Pharmacodynamics

### Pharmacokinetics.

An oral dose of 100–200 mg of doxycycline is almost completely absorbed in the duodenum and stomach and is detectable in the blood 15–30 minutes after administration. Following a 200 mg oral dose, peak concentrations of ∼2.6 μg/mL are reached at ∼2 hours, but this may vary as gastrointestinal (GI) absorption rates can differ among individuals.^3^,^36^,^37^ Doxycycline, being highly lipophilic, is readily transported across cell membranes, resulting in widespread distribution in body tissues and fluids.^37^ Doxycycline binds to serum proteins, localizes in the bone marrow, liver, and spleen, crosses the placenta, and is excreted in breast milk.^3^ Tetracyclines also form tetracycline-calcium orthophosphate complexes in sites of calcification such as developing teeth and bone.^3^ The bioavailabililty of the doxycycline derivatives, the monohydrate free base and the hydrochloride salt (hyclate), has been shown to be equivalent.^37^

According to the FDA, the absorption of doxycycline “is not markedly influenced by simultaneous ingestion of food and milk,” despite the reduced absorption observed with other tetracyclines. However, more recent studies on this subject, while limited, have shown that the absorption of doxycyline is affected to some degree by food and milk, but the clinical consequence of this is not well understood. Food has been shown to decrease serum levels of doxycycline from 4.4 to 4.0 μg/mL, but this has not been shown to be clinically significant.^3^,^37^ Milk decreases the absorption of tetracyclines because of chelation between the calcium in the milk and tetracyclines, however the magnitude of this decrease varies between different tetracycline preparations, and the data for doxycycline is limited.^38^,^39^ One single-dose, cross-over study administered doxycycline with either water or milk to nine volunteers and found that milk decreased the peak plasma drug concentration by 24% and that the area under the plasma concentration-time curve from 0 to 32 hours decreased by 35%.^39^ How this decrease in bioavailability translates clinically is not known. Furthermore, doxycycline is a lipophilic drug and it is not known if the fat content of the food or drink affects its absorption. In these studies, the fat content of the food and milk ingested was not discussed. Therefore, food taken with doxycycline to prevent gastrointestinal side effects is not expected to decrease efficacy of the drug; however, there is some limited data that suggests that milk reduces the bioavailability of doxycycline, which may have a negative impact on drug efficacy as in other tetracyclines. Because of absent better data, it would seem prudent to caution individuals to avoid milk and other dairy products when ingesting their daily doxycycline. Separation of the ingestion of doxycycline and ingestion of milk by 2–3 hours (time for absorption and peak concentration) should be satisfactory.

Unlike other tetracyclines, excretion of doxycycline occurs primarily by the gastrointestinal tract and to a much lesser extent by the kidneys.^40^–^42^ Most of a doxycycline dose diffuses from the serosal to the mucosal side of the small bowel. Doxycycline is chelated to an inactive form intraluminally and is excreted.^41^,^43^ A very small portion is also concentrated by the liver in bile for excretion in feces.^41^,^43^ Serum half-life of doxycycline (15–25 hours) is not affected by impaired renal function or hemodialysis, and in patients with renal failure, all excretion of doxycyline occurs by the gastrointestinal route of excretion.^42^,^43^

There are limited to no data on sex, age, body weight, or race differences in the pharmacokinetics of doxycycline. One study found no differences between males and females (*N* = 14 males and 14 females).^44^

### Pharmacodynamics.

Although it is known that doxycyline is a blood schizontocide,^45^ the exact mechanism of action is not well defined. In *P. falciparum*, doxycycline has been observed to block the expression of apicoplast genes, leading to nonfunctional apicoplasts in subsequent progeny, and impeding the development of viable parasites.^46^ It is also thought that doxycycline's antimalarial actions may be similar to its bacteriostatic actions of binding to ribosomal subunits and inhibiting protein synthesis, but this has only been observed in supra-pharmacologic doses.^47^ Doxycycline has also been observed to have some degree of pre-erythrocytic activity in *P. falciparum*.^16^ Of 55 men given a doxycycline prophylaxis regimen that ended 3 days after travel, 13 developed *P. vivax* and none developed *P. falciparum*.^16^ Two other studies observed breakthroughs to doxycycline prophylaxis for *P. falciparum* between 2 and 12 weeks after administration of medication and for *P. vivax* between 4 and 9 weeks.^14^,^15^ There are no data on the causal activity of doxycycline for the other species of malaria. On the basis of these studies, the recommended duration of the post-exposure course of doxycycline is 4 weeks. Doxycycline has no effect on the hypnozoites of the *Plasmodium* species.^16^,^45^ Because of this, when treating patients infected with species such as *P. vivax* and *P. ovale* with quinine and doxycycline, a 2-week course of primaquine must be added to kill hypnozoites.^48^ There is very limited evidence on doxycycline's effect on gametocytes. Only one study was found that looked at gametocytemia and doxycycline. In this study, *P. vivax* gametocytemia increased after treatment with doxycycline (from 32% pre-treatment to 44% immediately post-treatment) with a median gametocyte clearance time of 62 hours.^49^ Although gametocytemia has no clinical implications, malaria in an individual being treated with doxycycline may still be transmitted through mosquitoes.

There is no global consensus on the threshold of susceptibility for use in *in vitro* susceptibility testing for doxycycline. Traditionally, culture-derived or field *P. falciparum* isolates are incubated with varying concentrations of drug and inhibition of replication is measured by several methods to yield a 50% inhibitory concentration (IC50) at 48, 72, or 96 hours. The assay conditions and the drug mechanism of action can markedly affect results.^50^ Frequently, the incubation time for these assays is one cycle of parasite replication or 48 hours, but for doxycycline at least 72 hours and preferably 96 hours is needed, depending on the assay method, to obtain accurate results. The increased incubation time is required because doxycyline has been shown to exert its effect during the first 48 hours, but is only detectable in second generation parasites near the 96 hour time point.^46^,^51^,^52^ However, most studies have been limited to 48-hour assays. One study in French Guiana suggested an IC_50_ cutoff level of 9.6 μM for a 48-hour assay and noted that after both quinine and doxycycline were recommended as first-line malaria treatment in 1995, there was an increase of mean IC_50s_ from 9.6 μM in 1996–1999 to 13.1 μM in 2005.^53^ Another study examined *P. falciparum* samples of French patients who traveled to various African countries between 1997 and 2006 using a 42-hour assay and found an overall mean IC_50_ of 9.3 μM with country-specific means ranging between 6.2 and 11.1 μM.^54^ The findings of these two studies found many samples with IC_50s_ above the suggested 9.6 μM threshold. However, these findings are not consistent with the relative dearth of reporting of doxycycline failure (when taken appropriately either as prophylaxis or treatment) in the literature. Another study further identified possible molecular markers for decreased *in vitro* susceptibility to doxycycline defined as an IC_50_ of 35 μM.^55^

Resistance of *P. falciparum* to doxycycline is not described but breakthroughs in prophylaxis have been associated with inadequate doses,^14^ possibly inadequate serum levels,^11^ and poor compliance.^56^,^57^ A clear relationship between the development of clinical symptoms with confirmed *P. falciparum* parasitemia and a serum or plasma level of doxycycline or a predictive IC_50_ in a well-qualified *in vitro* susceptibility assay remains to be determined. More studies are needed to examine emergence of doxycycline resistance and establish a clinically relevant susceptibility threshold.

## Adherence

Adherence to daily doxycycline throughout the period of exposure to malaria and for 4 weeks following exposure is vital in achieving full effectiveness of doxycycline as a malaria prophylactic agent. The effectiveness of doxycycline among persons in whom adherence was not enforced was compared with persons in whom compliance was enforced. Effectiveness was only 89% (78–96%) for all types of malaria compared with 99% (94–100%) in soldiers in whom adherence was enforced.^13^ Because of doxycycline's short half-life and the mechanism of action (asexual parasite killing at the end of two cycles of erythrocytic replication or 96 hours) relative to that of weekly medications, missing a few doses of doxycycline would have greater consequences than being a few days late for a medication with a long half-life.

Most studies suggest that adherence to daily doxycyline is more challenging than adherence to a weekly medication. One study reported that full adherence to doxycycline daily (81%, *N* = 52) was lower than that for weekly mefloquine (98%, *N* = 344) among United States troops in Somalia; however, it was not discussed whether administration of one or both medications was supervised.^19^ Another study in Thai soldiers did not enforce or observe drug administration and reported that adherence (at least 75% of the time) occurred in more than 70% of men given a weekly drug (Maloprim) and in only 50% given doxycycline daily.^17^ Survey-based studies of travelers or military personnel have found adherence rates for doxycycline prophylaxis to be between 70% and 84%. A decrease in adherence over time has also been demonstrated. In Australian troops, adherence to daily doxycycline decreased from 60% at 2 months to 44% at 4 months.^21^ Only one study of French soldiers in sub-Saharan Africa comparing daily doxycycline to weekly chloroquine-proguanil (CP) found that a decrease in adherence occurred over time in both groups regardless of regimen.^58^ Therefore, studies seem to show that short-term adherence to daily prophylaxis is more challenging than for a weekly medication, but long-term adherence decreases for both daily and weekly prophylactic regimens.

## Safety and Tolerability

### General (Tables 3^10^,^13^–^16^,^19^,^20^,^28^,^57^,^58^,^61^–^64^ and 4,^10^,^12^–^15^,^19^,^21^,^28^,^57^,^58^,^61^–^64^).

Doxycycline is generally well tolerated with GI complaints and photosensitivity among the most common adverse events (AEs). Most available information on AEs in doxycycline are from studies examining doxycycline at prophylactic doses (100 mg/day), and there is limited information on AEs in doxycycline at treatment doses. At prophylactic doses of doxycycline, reports of mild and moderate AEs vary widely among studies from 0.6% to 84% of the study population.^13^,^20^,^21^,^59^–^61^ This variation can be explained in part because of a high background rate of non-specific symptoms experienced by both non-immune travelers and semi-immune study subjects in malaria endemic areas, differing definitions of mild and moderate AEs, use of different salts and formulations of doxycycline, and different study populations.

Reports of AEs of doxycycline at treatment doses (100 mg twice a day) are very limited because studies often examine malaria treatment with doxycycline in combination with another drug such as quinine, and it is difficult to determine if symptoms are related to doxycycline alone, malaria, the accompanying drug, or because of a drug-drug interaction. One randomized, open-label study comparing treatment of malaria with doxycycline and quinine versus artemether-lumefantrine found that at least one AE was reported in 93.5% of patients treated with quinine-doxycycline and 89.2% of those treated with artemether-lumefantrine; statistically similar proportions.^28^ No serious AEs were noted in both groups.^28^

### Gastrointestinal adverse events.

Gastrointestinal symptoms are usually mild to moderate and include nausea (4–33%) and abdominal pain (12–33%).^13^–^15^,^19^,^20^,^58^,^62^,^63^ Nausea has been noted to be more common when doxycycline is ingested without food.^13^,^21^ Vomiting (4–8%) and diarrhea (6–7.5%) are less commonly reported.^13^–^15^,^62^,^63^ The GI symptoms requiring a change in medication have been reported infrequently (0.2–6.4%).^12^,^20^,^21^

Doxycycline and other tetracyclines are believed to be the most common cause of drug-induced esophageal disorders.^64^,^65^ It is thought that long transit times of the pills in the esophagus contributes to mucosal inflammation and that ingestion of a sufficient amount of fluid, taking the pill while in an upright position, and avoiding taking the pill before bedtime could decrease the transit time and prevent inflammation.^65^,^66^ Doxycycline has been implicated in esophagitis; among United States military personnel returning from Somalia, 4 of 499 (0.8%) who took daily doxycycline developed odynophagia with dehydration requiring hospitalization.^57^ One other study, a shipboard survey, found esophagitis in 1 of 188 persons taking doxycyline prophylaxis.^63^

The effect of various doxycycline salts and formulations such as oral suspensions, tablets, capsules, and enteric-coated tablets are likely quite significant in their ability to cause GI adverse events. Gelatin capsules have been observed to be more sticky than tablets, delaying transit time or even adhering to the esophageal lining, and this is affected by the amount of fluids ingested with the pills.^65^,^67^ Enteric-coated formulations are available and may decrease GI side effects. One double-blind, placebo-controlled, multiple-dose cross-over study compared the incidence of GI side effects of enteric-coated doxycycline hyclate pellets in capsules (Doryx, Parke-Davis, Morris-Plains, NJ) and doxycycline hyclate powder in capsules (Vibramycin, Pfizer, New York), the enteric-coated capsule formulation produced statistically significantly fewer episodes of nausea, vomiting, abdominal discomfort, and decreased appetite than did the non-enteric-coated capsules.^68^ Differences in GI adverse events between doxycycline salts may exist. In the majority of studies in which doxycycline has been used for malaria prophylaxis, the source of doxycycline, the salts used, and the formulation was not reported. Because the exact active drug ingredient and the formulation used seems to affect the incidence and severity of GI adverse events, rates between studies are difficult to compare. However, one study^12^ examining efficacy of doxycycline hyclate in the French Army noted a 6% withdrawal rate caused by GI AEs and attributed this to the hyclate salt acidity and capsule form. Fewer withdrawals caused by GI AEs (1.5% and 4.9% at two study sites) were observed in a follow-up study^58^ looking at tolerability of doxycycline monohydrate. Although data are limited, it appears that enteric-coated hyclate formulations and monohydrate salts are better tolerated in terms of GI AEs than standard hyclate capsules or tablets.

### Photosensitivity.

An erythematous rash in sun-exposed areas has been reported to occur in 7.3–21.2% of persons^16^,^19^,^57^ taking doxycycline for malaria prophylaxis. One study examined the tolerability of various malaria prophylactic regimens and found that doxycycline did not cause a significantly higher percentage of all skin events (photosensitivity not specified) when compared with other antimalarials. The rash resolves upon discontinuation of the drug. A study of 106 acne patients taking daily doxycycline found a high incidence (35.8%) of light-sensitive rash and suggested that the phenomenon is dose related; 20% of patients taking 150 mg/day of doxycycline developed a light-sensitive rash, whereas 42% of those taking 200 mg/day were affected.^69^ It is suggested that patients with lighter complexions (Fitzpatrick skin types 1 and 2) may be more susceptible to doxycycline photosensitivity than patients with darker skin pigmentation.^70^ Both ultraviolet A (UV-A) and ultraviolet B (UV-B) radiation have been implicated.^71^ Although the mechanism of the phototoxic reaction has not been fully elucidated, it is believed to be mediated by excited-state singlet oxygen and free radicals after irradiation with UV-A radiation, thereby causing selective injury to mitochondria, within which doxycycline and other tetracyclines are localized.^72^ Patients developing photosensitivity should be advised to stop taking doxycycline. Avoiding sun exposure, wearing protective clothing, and using a broad spectrum (protection against both UV-A and UV-B radiation) sunscreen can lower the risk of photosensitivity.^73^,^74^

### Vulvovaginitis.

Antibiotics such as tetracyclines are believed to suppress vaginal bacterial flora, resulting in overgrowth of candida.^75^ A recent *in vitro* study found that tetracycline may also enhance *Candida albicans* virulence factors, but the authors note that it is still unclear as to what degree this contributes to clinical vaginitis.^76^ Increased colonization with *C. albicans* after taking tetracyclines has been shown.^75^,^77^,^78^ After a 3-week course of tetracycline, one study observed that the percentage of women with vaginal cultures positive for *C. albicans* increased from 7% to 24%.^77^ Another study had similar findings for both tetracycline and minocycline.^78^ Despite this increase in yeast colonization, both studies found that only a small percentage of women with positive candidal vaginal cultures had symptoms of vulvovaginitis.^77^,^78^ In fact, other studies have found that only a small percentage of women develop clinical vulvovaginitis after taking tetracyclines.^10^,^79^ In women taking tetracycline for acne, symptomatic vaginitis was observed in 5%.^79^ In another study, only one of 21 (4.8%) women on daily doxycycline withdrew from the study because of repeated episodes of vaginitis.^10^ Another study did not find an increased risk for vaginal candidiasis with doxcycyline.^61^ Of note, reporting of vaginitis as an adverse drug reaction specifically to doxycycline when taken as malaria prophylaxis is limited because vaginitis is often included under the category of non-specific skin reactions and many of the early studies were done in male military personnel. Women taking doxycycline who have a predisposition to candidal vulvovaginitis, such as those with diabetes mellitus, past history of recurring vulvovaginitis, and those taking oral contraceptives, should consider carrying a self-treatment course of antifungals.

### Other AEs.

There are other AEs that have been reported in association with tetracyclines. These include case reports of benign intracranial hypertension with tetracycline, minocycline, and doxycycline, but this has been observed to resolve upon discontinuation of the medication and has not been described in someone taking doxycycyline for malaria prevention.^80^–^82^ Skin hyper-pigmentation has been reported in the dermatological literature associated primarily with minocycline, and seems to mainly occur with long-term use and usually fades slowly after discontinuing treatment.^83^–^88^ Only one case report mentions skin hyper-pigmentation associated with doxycycline in a patient who had taken 1 g dose of doxycycline daily for 12 years.^89^ Other AEs mentioned in case reports associated with tetracyclines include postinflammatory elastolysis and tooth discoloration in an adult with long-term use (12 years).^90^,^91^ Central nervous system symptoms such as vertigo and ataxia have been more frequently associated with minocycline as compared with doxycycline because of minocycline's greater lipid solubility.^92^ *Clostridium difficile*-associated diarrhea (CDAD) has been reported in three patients taking doxycycline for malaria prophylaxis for between 2 and 6 weeks.^93^ A more recent case-control study examining antibiotic use and subsequent CDAD found doxycycline to be protective, however, this study did not examine the duration and dose of antibiotic therapy.^94^

### Comparative tolerability.

There are varying reports of the comparative tolerability of doxycycline. Several non-randomized survey-based studies in military personnel have found a higher occurrence of photosensitivity, GI symptoms, headache, and light-headedness with doxycycline as compared with mefloquine, whereas others have found that doxycycline is better tolerated than mefloquine.^19^,^20^,^57^,^63^,^64^ The type of doxycycline salt and formulation used in these studies were rarely reported, making it difficult to distinguish if the comparative tolerability of doxycycline to other antimalarials was affected by the type of doxycycline preparation used. One randomized, open-label trial found that while more participants taking doxycycline monohydrate withdrew from the study, their rates of adherence were higher compared with those taking chloroquine/progruanil.^58^ However, another randomized, open-label trial found doxycycline (unspecified salt) to be tolerated as well as chloroquine.^15^

The strongest evidence for doxycycline's relatively good tolerability is found in three randomized, double-blinded trials. Two of the studies found little or no difference in the frequency of AEs between doxycycline (unspecified salt) and mefloquine, and all AEs were mild and transient.^13^,^62^ In the third study, doxycycline monohydrate, atovaquone/proguanil, mefloquine, and chloroquine plus proguanil were compared (*N* = 623 evaluable persons).^61^ Severity of AEs was graded on a scale of 1–4 with 1 being trivial and 4 requiring hospitalization. Although no significant differences were seen between groups for mild (grade 1) or severe (grade ≥ 3) AEs, moderate AEs (grade = 2) differed significantly between groups. Moderate AEs were reported by 45% of chloroquine/proguanil users, 42% of mefloquine users, 33% of doxycycline monohydrate users, and 32% of atovaquone/proguanil users. Although not statistically significant, doxycycline monohydrate had the lowest incidence of severe adverse events (6%) of all regimens. Of these severe events, one-third was skin reactions. There were no significant differences in the rates of skin reaction or GI events between any of the regimens. When compared with the other medications, doxycycline monohydrate had an intermediate proportion of withdrawals, but this proportion did not differ significantly between the treatment arms. The authors concluded that of all four regimens, doxycycline monohydrate was one of the best tolerated.^61^ Overall, the evidence suggests that doxycycline is tolerated as well as other malaria prophylactic regimens.

## Contraindications

Doxycycline is contraindicated in anyone with a history of hypersensitivity to any of the tetracyclines.

### Duration of Use.

There are very limited studies on the use of doxycycline for malaria prophylaxis for a prolonged period of time. The current FDA product insert approves use for up to 4 months.^95^ One study surveyed 600 military personnel in Cambodia who took prophylactic doses of doxycycline for 12 months and 900 men deployed to Somalia for 4 months.^21^ Doxycycline was well tolerated; only 7 (0.6%) in Cambodia and 15 (1.7%) in Somalia discontinued medication because of adverse events related to GI problems and photosensitivity. Another study surveyed 228 U.S. Peace Corps volunteers who had taken doxycycline prophylaxis for an average of 19 months.^64^ Of these, 45 (20%) changed medications caused by adverse events such as GI symptoms, pruritic skin reactions, photosensitivity, and vaginal yeast infection. Compared with mefloquine in which 28% of respondents changed medication caused by adverse events, doxycycline was better tolerated. Of note, in these two studies, the types of adverse events reported were the same as those in persons using doxycycline for shorter periods of time. However, both studies used post-trip surveys and did not have information on adherence for all participants.

Although long-term safety and tolerability studies have not been performed with doxycycline as a malaria chemoprophylaxis, similar drugs in the same class, tetracycline and minocycline, have been used in equivalent doses (less than 1 g/day for tetracycline, 50 mg/day to 100 mg twice a day for minocycline) for a few months to several years in the treatment of various dermatological conditions. This suggests that doxycycline can be used for long-term malaria chemoprophylaxis. When used at this dosage, tetracyclines were found in some studies to cause no abnormalities in renal and hepatic function when taken for as long as 5 years and in one study, for as long as 13 years.^96^–^98^ There have, however, been several case reports of minocycline-associated autoimmune hepatitis and systemic lupus erythematosus-like syndrome in persons who used minocycline at 100 mg or more daily for more than 1 year.^99^–^103^ Of note, doxycycline users were not found to have an increased risk of hepatotoxicity in one matched case-control study.^104^ Scleral, dental, and dermal hyperpigmentation has also been reported in patients taking long-term minocycline as early as 1 month after initiation of treatment,^86^,^87^,^105^,^106^ but this hyperpigmentation has been shown to fade after discontinuation of the medication.^86^,^87^,^105^,^106^ In a 2-year, double-blind comparison of minocycline (100 mg twice a day) and hydroxychloroquine for the treatment of rheumatoid arthritis, only 10% of patients withdrew because of rash or GI symptoms.^106^ Overall, these case reports and studies observed rare to occasional adverse events associated with long-term use of other tetracyclines.

## Therapeutic Index and Overdose

Tetracycline antibiotics are relatively non-toxic. Exceeding the recommended dosage of doxycycline may result in increased incidence of ADRs. There has been one case report of an individual who took 1 g of doxycycline/day for 12 years.^89^ He was found to have hepatocellular necrosis with cholestasis, nephrotoxicity, leucopenia with anemia, skin hyperpigmentaion, and intermittent supraventricular tachycardia with sporadic Wenckebach heart block. He was not found to have any of the common doxycycline side effects such as GI symptoms or photosensitivity. After discontinuation of the drug, most of these symptoms resolved with the exception of residual cutaneous hyperpigmentation, relapsing cardiac arrythmias, and increased bilirubin.^89^ In the case of overdosage, the medication should be discontinued and supportive measures and symptomatic treatment instituted.^95^

## Drug Interactions

Doxycycline was introduced into clinical practice long before the recognition of drug-drug interactions and without currently required pre-licensure *in vitro* or *in vivo* studies.^107^ The potential for drug-drug interactions with doxycycline based on a full panel of *in vitro* studies with phase I metabolizing enzymes (cytochrome P450), phase II enzymes, and transporter mediated drug interactions has not been reported. On the basis of a single *in vitro* study showing that doxycycline inhibits the metabolism of quinine to 3-hydroxyquinine, the major metabolic pathway known to be enzymatically catalyzed by CYP 3A4, doxycycline is thought to be a modest inhibitor of CYP 3A4. Because CYP 3A4 is the most common phase 1 enzyme involved in drug metabolism, the spectrum of potential drug-drug interactions is wide and is not well defined. Furthermore, *in vitro* interactions do not always predict clinically significant effects. Information on drug-drug interactions with doxycycline is based on clinical observations and predictions are based on limited data accumulated over the last 40 years of clinical use, as described below.

Tetracyclines can chelate divalent and trivalent cations in certain medications that may cause a decreased absorption of the tetracycline and the medication when given concurrently.^3^ These medications include antacids with aluminum, calcium or magnesium, laxatives with magnesium, and oral iron preparations. Other medications decreasing absorption of tetracyclines include antidiarrheal agents containing kaolin, pectin, or bismuth subsalicylate. Medications such as these should be taken a few hours before doxycycline.^3^,^108^

Although a possible interaction with oral contraceptives (OCs) has been cited in older literature,^3^ current evidence has not supported this information.^109^–^111^ The overall OC failure rate in women who were also treated with antibiotics was found to be 1.2–1.6%, and this failure is thought to occur in individuals in whom plasma concentrations of ethinyl estradiol are decreased.^110^ Furthermore, the failure rate observed in concurrent administration of OC and antibiotics is well within the 1–3% failure rate observed with typical OC use.^110^,^112^ Therefore, recent evidence suggests that doxycycline can be used concurrently with OCs without leading to a higher rate of contraceptive failure than would be expected among OC users not currently taking antibiotics.

Tetracyclines may potentiate the effect of oral anticoagulants by impairing the use of prothrombin and decreasing vitamin K production by intestinal bacteria.^3^ The literature is limited, and there has been only one case report of a woman well controlled on warfarin, who developed an increased international normalized ratio and retroperitoneal bleeding when doxycycline was administered.^113^ It was hypothesized that in addition to the aforementioned mechanism, competition with protein binding or the occurrence of an inhibitory mechanism by the cytochrome P450 system could be factors.^113^ Patients on oral anticoagulants should have their prothrombin times monitored closely and dosage adjusted as needed.^3^

As a bacteriostatic drug, tetracyclines may interfere with the bactericidal action of aminoglycosides and penicillin^3^,^36^ but this has only been reported *in vitro*,^114^ and may not be of clinical significance. Doxycycline can be used concurrently with ciprofloxacin and azithromycin, two antibiotics commonly given to travelers for self-treatment of traveler's diarrhea.

Fatal nephrotoxicity has been reported in patients receiving methoxyflurane anesthesia and tetracycline.^115^–^117^ Although this has not been observed with doxycycline, specifically, concurrent use of tetracyclines and methoxyflurane anesthesia is not recommended.

Concurrent use of digoxin and oral antibiotics such as tetracyclines may increase serum digoxin concentrations in some individuals. It is believed that the tetracycline alters gut flora, diminishing digoxin conversion to inactive metabolites, resulting in increased serum digoxin concentrations.^118^,^119^ However, the data is limited and there have been no reports of toxicity caused by concurrent administration of digoxin and tetracyclines.

Barbiturates, carbamazepine, and phenytoin are believed to decrease the half-life of doxycycline by inducing microsomal enzyme activity.^41^,^120^ It has been suggested to double the doxycycline dosage (take twice daily) in those taking antiepileptics.^120^

Theoretically, antibiotics such as doxycycline could decrease the effectiveness of the oral typhoid vaccine Ty21a by preventing replication of the live attenuated *Salmonella* Ty21a bacteria used in the vaccine. However, there is no experimental data in animals or humans to support this position. Nevertheless, most experts advise not giving doxycycline within 24 hours of the oral typhoid vaccine.^1^ Because the immunization schedule for oral typhoid vaccine is recommended to be completed at least 1 week before potential exposure to *Salmonella typhi*,^121^ there should be no opportunity for interaction between the oral typhoid vaccine and doxycycline in most travelers.

There are no known drug interactions between doxycycline and antiretrovirals.

## Special Populations

### Children.

Doxcycycline is not recommended for children < 8 years of age because tetracyclines have been shown to cause permanent discoloration of the teeth.^122^,^123^ Tetracyclines form a complex with calcium orthophosphate that becomes incorporated into bones and teeth undergoing calcification.^124^ Tooth discoloration is permanent because remodeling and calcium exchange do not occur after calcification is completed. Some believe teeth staining is related to the total dose of tetracycline^122^,^123^ while others argue that it is caused by repeated courses.^125^,^126^ It is also thought that timing of tetracycline administration during tooth development, especially during early dentin formation, determines the degree of discoloration.^126^ By 8 years of age the dentin and calcium-rich enamel has formed on almost all teeth (except wisdom teeth), and therefore doxycycline can be taken after this age. There are some who recommend that the lower age limit for taking doxycycline should be 12, when most children have had their primary teeth replaced by their adult dentition. Doxycycline, however, may cause less tooth discoloration than other tetracyclines because it has a lower binding affinity to calcium.^127^,^128^ In fact, one study found that a short course of doxycycline given to children for Rocky Mountain spotted fever did not cause clinically significant staining of teeth.^129^

Of note, doxycycline is considered the drug of choice for treatment of infections in which the risks of tooth discoloration is outweighed by the benefit of disease treatment, such as presumed or confirmed rickettsial infection, ehrlichiosis, murine typhus, or inhalational anthrax.^129^–^135^ Because there are other drug alternatives for treatment and prophylaxis of malaria in children < 8 years of age, use of doxycycline is not recommended.

The formation of the tetracycline and calcium orthophosphate complex in developing bone is thought to cause growth retardation, but the evidence is limited. Decreased long bone growth rate has been observed in premature infants given tetracycline but is reversible upon discontinuation of the drug.^136^,^137^ There are no studies showing tetracycline-induced growth retardation in term infants or older children.

### Pregnant women.

Doxycycline is a category D drug; therefore, the possibility of pregnancy in a woman of childbearing age must be addressed before administration of this drug.^124^ Doxycycline is not recommended for malaria prevention or treatment in pregnancy, except for the treatment of life-threatening multidrug-resistant *P. falciparum* when no other treatment options are available. No congenital malformations have been associated with its use,^124^,^138^,^139^ and some limited evidence suggests that use of doxycycline in the first trimester might not cause adverse events in the fetus.^140^,^141^ However, tetracyclines cross the placenta and have been observed to cause discoloration of teeth when given during the second or third trimester of pregnancy.^142^,^143^ Because deciduous teeth begin to calcify at 5–6 months gestation, use of tetracycline during this time can result in staining.^124^ Additionally, on the basis of observed retardation of bone growth in premature infants,^137^ there are concerns of inhibition of bone growth in the fetus.

Tetracyclines have also been associated with fatal liver necrosis in pregnancy.^138^,^144^,^145^ These reports, however, are rare and have usually involved higher doses of doxycycline than those used for malaria prophylaxis and treatment.^146^

For women planning on becoming pregnant, there is no need to wait a specific period of time after doxycycline use before attempting to conceive, based on the short half-life (15–25 hours) of the drug.^3^,^36^ If women or their health care providers wish to decrease the amount of doxycycline in the body before conception, after 6 half-lives (3–6 days) only 2% of the doxycycline remains in the body.^147^

### Breastfeeding.

Data are extremely limited on the use of doxycycline in breastfeeding women. Doxycycline is excreted in breast milk at concentrations 30–40% of that found in maternal blood,^138^,^148^ and it is thought that the drug's absorption by infants is inhibited by the calcium in the breast milk. Furthermore, tetracycline was undetectable in breastfed infants whose mothers were taking tetracycline.^149^ Tetracycline has also been assessed by the American Academy of Pediatrics to be compatible with breastfeeding.^149^ Although data for doxycycline and breastfeeding are limited, most experts feel that the theoretical possibility of teeth discoloration and bone growth inhibition in the nursing baby would be remote. Concerned mothers or doctors may consider alternative drugs such as mefloquine, or for mothers nursing babies weighing more than 5 kg, atovaquone/proguanil.

### Medical conditions.

In patients with impaired renal or hepatic function, the dose of doxycycline does not have to be adjusted because it is excreted as an inactive chelated product by a complex process of back diffusion in the small intestine.^150^ Additionally, the clearance of doxycycline has been shown to be similar to those with normal renal function and in those with renal failure.^151^

In patients with seizure disorders in whom mefloquine is contraindicated, doxycycline can be given as an alternative.^147^ However, antiepileptics have been noted to shorten the half-life of doxycycline and dosing adjustments must be made accordingly (see Drug Interactions section).

Doxycyline (Vibramycin) syrup contains metabisulfite, which can cause allergic reactions, including anaphylaxis and asthmatic episodes in susceptible individuals.^3^

Persons being treated for skin conditions with minocycline and who are in need of malaria prophylaxis should stop minocycline and start doxycycline. There is more data on the antimalarial efficacy of doxycycline compared with minocycline, therefore minocycline is not recommended as a replacement for doxycycline. Additionally, there have been case reports of autoimmune hepatitis in persons taking 100 mg or more of minocycline daily, whereas doxycycline has been observed to have less hepatotoxicity than other tetracyclines and less adverse events than minocycline.^100^,^101^,^104^,^152^ After completion of doxycycline for malaria prophylaxis, minocycline can be resumed.

## Cost

The drug cost for doxycycline varies by doxycycline salt, enteric formulation, or brand name versus generic. One article examining the cost considerations of malaria chemoprophylaxis looked at the average wholesale price (prices from 2006) of various antimalarial medications and found that generic doxycycline hyclate was much cheaper (100 mg, US $0.25 per dose) than generic doxycycline monohydrate (100 mg, $2.13 per dose) and that generic doxycycline monohydrate was much cheaper than the brand name (Vibratabs, doxycycline monohydrate 100 mg, $4.51 a dose).^153^ In this same work, the direct costs of various malaria chemoprophylaxis regimens were compared. For a 14-day stay, including pre- and post-exposure doses, the drug cost for prophylaxis was $11.00 for generic doxycycline hyclate compared with $84.64 for mefloquine and $113.39 for atovaquone/proguanil.^153^ Using generic doxycycline monohydrate brings up the cost of prophylaxis to that of mefloquine, and using brand name doxycycline monohydrate brings the costs up even further. To look at more recent costs, prices of antimalarials were examined online for the average wholesale price. A full course of malaria prophylaxis for a 14-day stay in a malaria-endemic area costs $18.49 for generic doxycycline hyclate capsules, $65.79 for generic doxycycline monohydrate capsules, $122.50 for Vibratabs, $406.35 for Doryx (enteric-coated doxycycline hyclate), $80.48 for mefloquine, and $159.94 for atovaquone/proguanil.^154^

## Exchanging Doxycycline for Other Antimalarials

There is very limited information on the best regimen for changing from doxycycline to other antimalarials, and recommendations for doing so are based on considering several issues such as drug-drug interactions, half-life differences, and differences in mechanism of action. If changing between atovaquone/proguanil and doxycycline, note that the current atovaquone-proguanil label lists a pharmacokinetic interaction of “concomitant treatment (of *P. falciparum* malaria) with tetracycline (with atovaquone) has been associated with approximately a 40% reduction in plasma concentrations of atovaquone,”^155^ however, there are no accessible published studies that support this. On the contrary, early *in vitro* studies of atovaquone plus doxycycline and atovaquone plus tetracycline showed potentiation between the two drugs, which was later confirmed in clinical studies by improved cure rates when compared with atovaquone alone.^156^–^158^ Therefore, if changing from doxcycyline to atovaquone/proguanil for malaria treatment or prophylaxis, doxycycline can be stopped and atovaquone/proguanil started at the same time. However, the recommended length of the post-trip course of prophylactic medications may change because the drugs target different stages of the *Plasmodium* life cycle. The Centers for Disease Control and Prevention (CDC) currently recommends that the atovaquone/proguanil is continued for up to 4 weeks after leaving the endemic area depending on when the change was made.^1^ If changing from atovaquone/proguanil to doxycycline at any time, a full prophylactic course of doxycycline should be taken (for the duration of the stay and 4 weeks after departure). Changing from doxycycline to other antimalarials such as chloroquine or mefloquine will be impacted by the difference in half-lives of these medications. Doxycycline's half-life of 15–25 hours is much shorter than that of chloroquine (6–60 days) and mefloquine (2–3 weeks).^1^ A change from doxycycline to chloroquine or mefloquine before travel would still require that chloroquine and mefloquine are taken 1 and 2 weeks before exposure, respectively, to attain effective blood concentrations before travel. It is not recommended to change from doxycycline to chloroquine or mefloquine during or after travel because effective blood concentrations will not be achieved for a few weeks, leaving the traveler unprotected during this time.

## Figures and Tables

**Table 1:** Efficacy of doxycycline for prophylaxis*

Country (population)	Study type	Sample size on doxycycline	Duration of prophylaxis	Efficacy (95% CI, if reported)
Kenya (semi-immune)^10^	Randomized, double-blind, comparators = azithromycin daily (*N* = 59), azithromycin weekly (*N* = 58), and placebo	55	10 weeks	Doxycycline: 92.6% (79.9–97.5%)
				Azithromycin daily: 82.7% (68.5–91.1%)
				Azithromycin weekly: 64.2% (47.1–77.1%) azithromycin weekly
Kenya (semi-immune, children aged 9–14)^11^	Randomized, double-blind, comparators = primaquine daily (*N* = 32), proguanil daily plus chloroquine weekly (*N* = 37), vitamin daily plus weekly mefloquine (*N* = 30), vitamin alone (*N* = 34)	32	11 weeks	Doxycycline: 84% (66–92%)
				Primaquine: 85% (68–93%)
				Mefloquine: 77% (55–88%)
				Chloroquine plus proguanil: 54% (25–72%)
Gabon and Central African Republic (non-immune)^12^	Randomized, open-label, comparator = CP (*N* = 270)	171	Travel: 4 months	Doxycycline: 97.0%
				CP: 98.9%
Irian Jaya (non-immune)^13^	Randomized, double-blind, comparator = MQ (*N* = 68) and placebo	67	13 weeks	Doxycycline: 99% (94–100%) overall, 98% (88–100%) for *P. falciparum*, 100% (90–100%) for *P. vivax*
				MQ: 100% (96–100%) overall, 100% (93–100%) for *P. falciparum*, 100% (91–100%) for *P. vivax*
Thai–Burmese border (semi-immune children aged 10–16 years)^14^	Randomized, open-label, comparators = half-dose doxycycline (*N* = 77), placebo	67	5 months	Full-dose: 5 cases/1070 person-weeks
				Half-dose: 20 cases/1176 person- weeks
Thai-Burmese border (semi-immune children aged 19015 years)^15^	Randomized, open-label, comparator = CQ (*N* = 93)	95	9 weeks	Doxycycline: 5 cases/597 person-weeks
				CQ: 31 cases/488 man-weeks
Papua New Guinea (PNG), Malaysia, Thailand (non-immune)^16^	Open-label, comparators = MQ (*N* = 40, PNG), doxycycline + PQ (*N* = 69, PNG), doxycycline + CQ (*N* = 125, Malaysia and Thailand)	60	6 weeks	Doxycycline (*N* = 60): 100% for *P. falciparum*, 96.7% for *P. vivax*
		55	3 weeks	Doxycycline (*N* = 55): 100% for *P. falciparum*, 76.4% for *P. vivax*
				MQ: 100% for *P. falciparum*, 90% for *P. vivax*
				Doxycycline + PQ: 100% for both *P. falciparum* and *P. vivax*
				Doxycycline + CQ:
Thai-Kampuchean border (non-immune)^17^	Randomized, open-label, comparators = half-dose doxycycline (*N* = 243) or phyrimethamine + dapsone (*N* = 123), adherence observed but not enforced	243	17 weeks	81% (Adherence 73% for both doxycycline groups)
Irian Jaya, Indonesia (non-immune)^18^	Randomized, double-blind, placebo-controlled, comparators: azithromycin (*N* = 148), placebo	75	20 weeks	Doxycycline: 97.1% (91.2–99.4%) overall, 96.3% (85.4–99.6%) *P. falciparum*, 98.0% (88.0–99.9%) *P. vivax*
				Azithromycin: 84.7% (75.5–90.7%) overall, 71.6% (50.3–83.8%) *P. falciparum*, 98.9% (93.1–99.9%) *P. vivax*

* CI = confidence interval; CP = chloroquine-proguanil; MQ = mefloquine; PQ = primaquine; CQ = chloroquine.

**Table 2:** Efficacy of doxycycline with or without quinine for treatment of *P. falciparum* malaria

Country (population)	Study type	Sample size/doxycycline dose	Duration of treatment	Cure rate (95% CI)
United States (mixture of immune and non-immune)^5^	Challenge, open-label	*P. vivax:*	4 days	0
		1/100 mg twice a day	5 days	0
			7 days	0
		1/100 mg twice a day		100%
		*P. falciparum:*		
		4/100 mg twice a day		
		9/100 mg twice a day		
West Malaysia (varied, children aged 2 months–8 years)^27^	Challenge, open-label	9/4 mg/kg daily	4 days	44.4%
		26/4 mg/kg daily	7 days	84.6%
Irian Jaya, Indonesia (non-immune)^26^	Randomized, open-label, comparator = CQ (*N* = 30) and doxycycline + CQ (*N* = 39)	20/100 mg twice a day	7 days	*P. falciparum*: 64.7% (42.0–87.4%)
				*P. vivax*: 33.3% (6.6–59.5%)
Brazil (semi-immune)^28^	Randomized, open-label, comparator = AL (*N* = 28)	31/100 mg twice a day + 500 mg quinine every 8 hours	5 days doxycycline	100%
			3 days quinine	
Pakistan (unknown immune status)^29^	Challenge, open-label	100/100 mg twice a day + 10mg/kg quinine every 8 hours	7 days doxycycline	100%
			3 days	

**Table 3:** Mild to moderate adverse events (AEs) associated with doxycycline prophylaxis and treatment*

Study location (design)	Sample size, dose, duration	Most frequent AE	Comparator	AE risk with doxycyline
Prophylaxis				
Kenya (RDBPC, semi-immune)^10^	55, 100 mg doxycycline hyclate daily, 10 weeks	Events:	Azithromycin daily	No difference between doxycycline and comparators
		Abdominal pain 49		
		Diarrhea 9	Azithromycin weekly	
		Vomiting 6		
		Vaginitis 5	Placebo	
Thailand (RDB, non-immune)^62^	119, 100 mg doxycycline daily, 5 weeks	Nausea 16.7%	MQ	No difference between doxycycline and MQ
		Headache 10.7%		
		Skin rash 10.7%		
		Dizziness 6.3%		
		Vomiting 4.4%		
Africa (observational, non-immune)^63^	188, 100 mg doxycycline daily, 4 days	Nausea 33.0%	MQ	Doxycycline group had significantly more nausea, abdominal pain, vomiting, drowsiness, headache, and dizziness than MQ goup.
		Abdominal pain 22.9%		
		Drowsiness 23.9%		
		Headache 19.7%		
		Vomiting 8.5%		
		Dizziness 7.4%		
		Rash 0.5%		
Irian Jaya, Indonesia (RDBPC, non-immune)^13^	67, 100 mg doxycycline daily, 13 weeks	Skin-related 33%	MQ Placebo	Doxycycline group had significantly fewer AE relative to MQ and placebo.
		Cough 31%		
		GI symptoms 24%		
		Headache 16%		
		Dizziness 9%		
		Insomnia 6%		
Thai-Burmese border (RPC, open-label, semi-immune, children aged 10–16 years)^14^	67, > 40 kg 100 mg doxycycline daily, < 40 kg 50 mg doxycycline daily, 5 months	Abdominal pain 16%	Doxycycline half-dose	Full-dose doxycycline more likely to have GI symptoms than half dose. Full-dose doxycyline less likely to have headache and dizziness than placebo.
		Nausea 12%		
		Diarrhea 8%		
		Vomiting 4%		
		Headache 34.3%		
		Dizziness 26.8%		
Thai-Burmese border (R, open-label, semi-immune, children aged 10–16 years)^15^	95, > 40 kg 100 mg doxycycline daily, < 40 kg 50 mg doxycycline daily, 9 weeks	Dizziness 27%	CQ	No difference between doxycycline and CQ
		Nausea 20%		
		Headache 17%		
		Fever 15%		
		Abdominal pain 8%		
		Vomiting 7%		
		Diarrhea 7%		
Papua New Guinea (open-label, non-immune)^16^	55, 100 mg doxycycline daily, 3 weeks	Gastrointestinal 24%	Doxycycline + PQ	No difference between doxycycline and doxycycline + PQ
		Photosensitivity 7.3%		
Somalia (observational, non-immune)^19^	52, 100 mg doxycycline daily, 7 weeks	Gastrointestinal 34.6%	MQ	Doxycycline had significantly higher rates of photosensitivity and gastrointestinal events than MQ.
		Photosensitivity 21.2%		
		Lightheadedness 19.2%		
Rwanda (observational, non-immune)^20^	28, 100 mg doxycycline daily, 2 months	Abdominal pain 33.3%	MQ	Doxycyline had higher rates of AE compared with MQ.
		Fatigue 23.8%		
Somalia (observational, non-immune)^57^	499, 100 mg doxycycline daily, varied time periods	Gastrointestinal 17%	MQ	No significant difference between doxycycline and MQ, except MQ had more insomnia or nightmares.
		Dizziness 10%		
		Insomnia or nightmares 9%		
		Sunburn 5%		
Sub-Saharan Africa (RDB, placebo run-in phase, non-immune)^61^	153, 100 mg doxycycline	Neuropsychological 24%	MQ	Doxycycline had less neuropsychological events than mefloquine, and less adverse skin events than CQ + proguanil. No increased risk of vaginal candidiases with doxycycline.
		Gastrointestinal 14%	CP	
		Skin itching, reddening 5%		
		Skin and vaginal itching, abnormal discharge 9%	Atovaquone and Proguanil	
Chad and Gabon (randomized groups, non-immune)^58^	275, 100 mg doxycycline monohydrate daily, 5 months	Events:	CP	Doxycycline had significantly less diarrhea, epigastralgia, mouth ulcers, urticaria, pruritis, rash, and sun sensitization than CP. No statistical difference in nausea and vertigo.
		Diarrhea 52		
		Epigastralgia 46		
		Nausea 30		
		Vertigo 14		
		Mouth ulcers 11		
		Pruritis 11		
		Skin rash 9		
		Sun sensitization 4		
Varied countries (observational, non-immune)^64^	228, 100 mg doxycycline daily, at least 6 months	Gastrointestinal 35%	MQ	Doxycycline had a higher proportion of skin, vaginal, and gastrointestinal events than MQ and CQ.
		Skin and vaginal 34%	CQ	
		Neuropsychologic 30%		
Treatment				
Brazil (R, open-label, semi-immune)^28^	31, 100 mg doxycycline twice a day for 5 days + 500 mg quinine every 8 hours for 3 days	Upper abdominal pain 41.4%	AL	Frequency of AE were similar for both doxycycline and AL.
		Nausea 34.5%		
		Vomiting 27.6%		
		Asthenia 27.6%		
		Headache 27.6%		

* MQ = mefloquine; GI = gastrointestinal; CQ = chloroquine; PQ = primaquine; CP = chloroquine-proguanil; AL = artemether/lumefantrine.

**Table 4:** Severe adverse events (AE) associated with doxycycline prophylaxis and treatment

Study location (design)	Sample size, dose, duration	Severe AE	Discontinued because of AE
Prophylaxis			
Kenya (RDBPC, semi-immune)^10^	55, 100 mg doxycycline hyclate daily, 10 weeks	Recurrent vaginitis 1	1 withdrew
Thailand (RDB, non-immune)^62^	119, 100 mg doxycycline daily, 5 weeks	None	None
Gabon and Central African Republic (non-immune)^12^	171, 100 mg doxycyline hyclate daily, 4 months	Gastrointestinal side effects 11	11 withdrew
Africa (observational, non-immune)^63^	188, 100 mg doxycycline daily, 4 days	Esophagitis 1	1 discontinued, requiring further evaluation and treatment
Irian Jaya, Indonesia (RDBPC, non-immune)^13^	67, 100 mg doxycycline daily, 13 weeks	None	None
Thai-Burmese border (semi-immune, children aged 10–16 years)^14^	67, 100 mg doxycycline daily, 5 months	None	None
Thai-Burmese border (R, open-label, semi-immune, children aged 10–16 years)^15^	95, > 40 kg 100 mg doxycycline daily, < 40 kg 50 mg doxycycline daily, 9 weeks	None	None
Rwanda (observational, non-immune)^19^	28, 100 mg doxycycline daily, 2 months	Abdominal pain 1	1 discontinued
Somalia, Cambodia (observational, non-immune)^21^	900, 100 mg daily, 4 months, all received primaquine eradication course after travel	Sun sensitization 13	15 discontinued
		Gastroinestinal problems 2	
Somalia (Observational, non-immune)^57^	499, 100 mg doxycycline daily, varied time periods	Odynophagia 4	4 discontinued, required hospitalization because of dehydration
sub-Saharan Africa (RDB, placebo run-in phase, non-immune)^61^	153, 100 mg monohydrate daily, 7–9 weeks	Skin and vaginal itching, abnormal discharge 4	5 withdrew, did not specify which AE
		Skin itching, reddening 3	
		Gastrointestinal 3	
		Neuropsychological 1	
Chad and Gabon (randomized groups, non-immune)^58^	275, 100 mg doxycycline monohydrate daily, 5 months	Gastrointestinal effects 9	15 withdrew
		Headache 5	
		Sun sensitization 1	
Varied countries (observational, non-immune)^64^	228, 100 mg doxycycline daily, at least 6 months	Gastrointestinal 11	Did not specify. Regimen changes caused by AE was lower among those taking doxycycline than those taking CQ or MQ.
		Skin and vaginal 9	
		Neuropsychologic 5	
Treatment			
Brazil (R, open-lab,^28^ semi-immune)^61^	31, 100 mg doxycycline twice a day for 5 days + 500 mg quinine every 8 hours for 3 days	Vomiting 1	1 withdrew
